# Lessons from Denmark for community-based governance of urban heat networks

**DOI:** 10.1007/s44327-026-00273-5

**Published:** 2026-06-10

**Authors:** Eirini Sampson, Zen Makuch

**Affiliations:** Centre for Environmental Policy, Imperial College London, Weeks Building, 16-18 Prince’s Gardens, London, SW7 1NE UK

## Abstract

Urban heat systems are pivotal to achieving climate-neutral, resilient, and equitable cities. Within the European Union’s Green Deal and Fit-for-55 frameworks, the decarbonisation of heating and cooling (H&C) - which accounts for over 60% of EU energy use - remains a critical challenge for sustainable urban transitions. District heating networks, often described as a “Swiss army knife for decarbonisation,” provide a key pathway for cities to integrate renewable electricity, recover urban waste heat, and strengthen energy resilience. Yet their development depends on governance models that embed justice, participation, and local empowerment. This paper presents findings from a Rapid Evidence Assessment of Denmark’s heating system, where two-thirds of domestic heat demand is met through district heat, and over 90% of networks are municipally or cooperatively owned. By analysing Denmark’s socio-technical and legal frameworks, the research identifies three enabling conditions for decarbonised urban heat transitions: (1) stable rule of law through the Heat Supply Act, which delegates planning powers to municipalities; (2) sustained financial mechanisms that support non-profit, community-based infrastructure; and (3) participatory ownership models that foster “innovative democracy” in energy governance. These insights show that urban heat networks function as public infrastructures of resilience when underpinned by strong and participatory governance. The Danish experience demonstrates a community-oriented governance model that offers transferable lessons for European cities seeking to decarbonise heating systems in a just and climate-compatible way.

## Introduction

The legally binding Paris Agreement envisages the creation of a *just transition* while limiting of global warming below 2 °C compared to pre-industrial levels to become climate neutral by 2050 [1]. The ‘European Union (EU) Climate Law’ has made large strides to meet its ambition to become the first carbon-neutral continent by 2050. To this end, by 2050, the EU is aiming to radically restructure its energy sector in the long-term under the EU Green Deal (2019). The decarbonisation of energy systems depends on the expansion of power generation from Renewable Energy Sources (RES), as pursued through the EU Renewables Directive [2], which progressively reduces reliance on conventional energy production [3]. However, Europe’s energy system remains highly centralised and hierarchically governed, with limited public participation and significant dependence on energy imports, contributing to energy insecurity and unaffordability - a vulnerability that was exposed by the Russian invasion of Ukraine in 2022, which left millions of Europeans facing energy poverty [4, 5].

The EU’s decarbonisation priorities are reflected in the new Commission’s mandate and the Draghi Report which called for the development of a ‘social contract’ to engage consumers in the energy transition [6]. The President’s letter to the new Commissioner for Energy and Housing outlined the need for a Citizens Energy Package, with the aim of increasing consumer participation and the protection of energy poor and vulnerable households [7]. In parallel, the non-programmability of RES - understood as the limited ability to dispatch or schedule energy production in line with demand due to weather-dependent variability - particularly at the local level of RES production, indicates the need for localisation and the empowerment of community prosumerism - that being consumers who acquire ownership of renewable energy infrastructure, generating their own energy - ensuring energy efficiency [3, 8–10]. Reflecting a new consumer-centric approach, the Renewable Energy Directive (RED-II) and the Internal Electricity Market Directive (IEMD) – under the prism of Fit-for-55 - established the legal basis for ‘prosumership’ – providing individual households or Renewable Energy Communities (RECs) with legal personality.

One of the key hurdles to implementing these EU legislative initiatives is the decarbonisation of the heating and cooling (H&C) sector. According to the IEA, only25% of European heat supplies are currently produced from renewable energy sources [11], with fossil fuels accounting for approximately 62% of supply [12, 13]. A key technology and sector in the decarbonisation of H&C in the EU is district heating, described by Euroheat and the IEA as a “Swiss army knife for decarbonisation” enabling the combined use of local renewable electricity, and excess heat from industrial and urban sources – including waste heat from data centres and wastewater, geothermal, and large-scale solar thermal – that cannot be efficiently utilised through individual household systems [14]. By supplying heat through shared networked infrastructure, district heat exploits economies of scale, higher linear heat demand density, and long asset lifetimes, making it particularly well-suited to urban and suburban contexts where concentrated demand enables efficient network operation [15]. However, district heat’s contribution to decarbonisation is not automatic: it depends on the heat sources used, the carbon intensity of the electricity grid, and whether network connection preserves or undermines incentives for building insulation – meaning its merits relative to individual solutions must be addressed contextually rather than assumed generically. District heat networks also exhibit natural monopoly characteristics, and whether these features serve the public interest depends entirely on governance arrangements: profit-oriented ownership risks limiting consumer agency, whereas public, municipal, or cooperative frameworks can support stable pricing, long-term planning and cross-subsidisation between users. In this sense, district heat is best understood as a shared urban infrastructure whose value for decarbonisation and community participation is shaped by the institutional conditions under which it is governed.

Over the past decade, research and pilot projects have demonstrated the potential of fourth- and fifth-generation district heating (4GDH and 5GDH) systems, including the use of decentralised energy substations, low-temperature networks, and digital control, to improve energy efficiency and support decarbonisation [16]. These newer network generations create technical conditions for active consumer participation, including peer-to-peer heat exchange and energy trading among connected users – possibilities that were not available in earlier district heat designs [17]. While such systems remain at an early stage of deployment and are not yet widely implemented across Europe, they represent a distinct opportunity to integrate energy communities into district heating networks, thereby empowering citizens as active participants in local energy systems. It is important, however, to distinguish these emerging 4GDH/5GDH-enabled models of participation from the longer-standing tradition of cooperative and municipal district heat ownership which has existed in countries such as Denmark and Germany since the early 1970s [9, 18–20]. While both reflect principles of community governance, the former involves technical participation in the network itself, whereas the latter concerns ownership and governance of the infrastructure.

Among European cases, Denmark stands out as a particularly mature and instructive case due to the central role of district heating within its national and urban energy system. Danish energy performance has been widely recognised, with the country ranking first in the Energy Trilemma Index in 2024, reflecting a balanced approach to sustainability, affordability, and security [21]. This performance is closely linked to two long-standing features of the Danish energy transition: a bottom-up governance approach that emerged in response to the 1973 oil crisis, and the systematic development of district heating as a core element of urban infrastructure [19, 22], with district heating supplying approximately two-thirds of household heat demand and being predominantly concentrated in urban and suburban areas [15]. While these features of the Danish energy transition are well-documented in the existing literature, how the specific legal, financial and governance arrangements underpinning Danish district heating systems can inform EU-level policy reform – and particularly the integration of energy communities into district heat systems in other Member States – remains unexplored. This paper addresses that gap.

While the EU has established robust legislative frameworks that adopt a multi-dimensional approach to energy law, their implementation remains highly compartmentalised, with limited interaction between key regulatory pillars of the energy transition. This fragmentation reinforces trade-offs between the three tenets of the Energy Trilemma while weakening the position of consumers both as end-users and as potential collective market actors.

Considering the Commission’s new mandate and the forthcoming revision of the EU Heating and Cooling Strategy, this paper aims to examine the integration of energy communities into the planning and operation of district heat networks as a governance pathway for decarbonising urban heat supply. Rather than assessing the technical deployment or expansion of heat networks per se, the paper focuses on the regulatory and institutional conditions under which district heating can support community participation, affordability, and long-term decarbonisation. Drawing on a Rapid Evidence Assessment of the Danish case, the study analyses how legal frameworks, financial arrangements, and cooperative ownership models have enabled the incorporation of community-based actors within district heating systems. The paper addresses the following research question: What regulatory and supporting policy reforms are required to integrate energy communities into the planning and operation of district heating networks in line with EU climate targets? The findings illuminate how Denmark’s governance and regulatory arrangements have enabled a rapid rollout and decarbonisation of district heating, and what lessons these arrangements hold for EU-level policy reform in relation to community integration into heat networks. The paper’s contribution lies not in revisiting the Danish model as such but in identifying the transferrable regulatory and institutional conditions that can support community-led governance of district heat within EU Member States.

The scope of this paper is limited to heating, rather than cooling which is often – misleadingly so – included in research despite the nuanced differences between district heating and cooling. Further, this research is limited to household consumers, excluding commercial and industrial users. While small and medium-sized enterprises (SMEs) are included within the scope of the Renewable Energy Directive (RED II), this study is limited to household consumers and excludes businesses, as incorporating commercial and industrial users would have significantly broadened the analytical scope and introduced distinct regulatory and contractual considerations beyond the focus of this research. The remaining of the article is split in the following structures. Section 2 presents the research’s background. Section 3 presents the methodology. Section 4 distils the lessons learnt and puts forth policy recommendations at national and EU levels from the rapid evidence review. Last, Section 5 presents a conclusion, lessons learnt, scope for further research and the implications for policy.

## Background

### The emergence of energy communities

Many countries are establishing enabling conditions for sustainable production and consumption practices alongside citizen empowerment. The transition includes energy decentralisation, perceived as a socio-technical process where institutional, technical, and socio-economic factors shape inclusivity in energy projects, resulting in the legal recognition and subsequent expansion of energy communities in several jurisdictions.

The European Energy Strategy and subsequent laws recognised the importance of consumers, community-driven initiatives, and municipalities as participating actors in the internal market to meet the EU’s energy law objectives and balance its Energy Trilemma. Under this Strategy, the Clean Energy Package (2019) introduced energy communities in two key legal instruments pertaining to the internal energy market and to renewable energy. These mandate Member States to adopt an enabling framework providing prosumers with legal personalities. Article 2[11] of the IEMD (EU) 2019/944 (2019) defines energy self-consumers (or Citizen Energy Communities (CECs)) [23]. This refers to a legal entity that is based on voluntary and open participation controlled by members or shareholders, whose primary aim is to provide socio-economic and environmental benefits to its members or the wider local area they operate in, based on the non-profit principle [10].

On the other hand, Articles 21 and 22, in RED-II, Directive (EU) 2018/2001 (2018), define RECs in stricter terms [2]. RECs must be controlled by their members in proximity of RES projects. Importantly, these two instruments provide that CECs and RECs can participate in several activities including energy sharing, distribution, and transportation. IEMD provides the regulatory framework that enable energy communities to have a level playing field when in competition with other market participants (Article 65). On the other hand, RED-II explicitly aims to facilitate the development of RECs (Article 22[2]) by mandating Member States to establish enabling mechanisms for the development of RECs in their jurisdictions. In addition, the REPowerEU plan put forward the shared political objective of achieving one energy community per municipality with a population of more than 10,000 residents by 2025 [24].

Energy communities are not only energy policy innovations but also emerging forms of urban governance that reconfigure relationships among citizens, municipalities and infrastructures. It is accepted that energy communities and decentralisation are a new agent in the energy mix. Their forms vary between Member States. Local authorities are taking a prominent role in setting up energy communities in collaboration with citizen companies and NGOs [25]. Other examples include communities of farmers and Small and Medium Enterprises (SMEs) cooperating with third parties such as project developers or energy suppliers. Energy community members transform from consumers to prosumers, reducing their dependence on a traditional, vertically integrated energy market [26].

The above Directives provide new rights and responsibilities for energy communities, while also implementing the creation of new mix dynamics between energy communities, Distribution System Operators (DSOs), regulators and consumers [26]. However, this development has not been without legal and political compromises, as stakeholders have raised concerns regarding energy communities foregoing regulatory free riding by being exempt from public duties including tax and network exemptions [27]. These responsibilities are not defined in either Directive, constraining the development and market participation of energy communities, and perpetuating end-user vulnerability to price increases. 

This is confirmed by [28] multi-agent model of urban microgrids which illustrated the benefits of energy community-trading. The subsequent market change positively affects consumer choice awareness while creating a niche that places end-users at the epicentre of technical innovation [26]. Energy communities have the potential to satisfy the goals outlined in the Paris Agreement and the European Green Deal, in enabling a just energy transition that leaves no one behind [3, 10, 29]. Their expansion involves the socio-technical transformation of citizens as active participants in the energy market, therefore expanding consumer empowerment and innovation while contributing to RES deployment growth [9]. For instance, Caramizaru and Uihlein’s report for the EU Commission cites a 2016 report commissioned by Greenpeace on the potential impacts of energy citizens in the EU, which estimated that half of EU citizens could own their own energy by 2050 [9].

While the relevant European Directives deliver some form of consumer-centric, legislative innovation, their provisions refrain from establishing a robust framework for enabling the expansion of energy communities. Therefore, while on the one hand, the Directives recognise the role that these entities may play in ensuring implementation of the so-called ‘social contract’ that is referenced in the Draghi Report, they do not provide details on the operative terms of such a 'contract' [30]. The Directives refrain from providing the “regulatory sandbox” proposed in the literature [31], and as a result, energy communities become vulnerable to the oligopolistic nature of the energy market. This is further exacerbated by varying Member State transposition levels which ultimately prevent the regulatory and practical differentiation of energy community structures from their traditional large scale energy market counterparts with whom energy regulators are familiar.

As the legislation does not provide the principles for governing energy communities, the literature has taken on the task. For instance, drawing on a dataset of 67 best-practice examples of consumer ownership Lowitzsch and colleagues argued that these governance structures must be broadly underpinned by some key principles [31, 32]. These include interconnectivity, complementarity, flexibility and bidirectionality based on collective control, integrated RES and energy efficiency. The authors propose the creation of “regulatory sandboxes” [31] for experimentation to find optimal preferential conditions for realising the abovementioned principles – which may be a more appropriate mode of loosely optimising the modes of governance for energy communities. This not only reconciles the heterogeneity of energy communities across Member States, but it also affords them with discretion in terms of implementing the above Directives based upon their national conditions. It is within this context of incomplete legislative architecture and unresolved governance principles that this case for Thermal Energy Communities (TECs) may be situated. 

### Thermal communities in the law and literature

Against this backdrop, the following examines how the above regulatory gaps take on particular significance in the context of thermal energy communities, where the distinct physical and legal characteristics of heat networks introduce governance challenges that existing EU frameworks are less equipped to address. In its 2023 recast, RED-III reflected the EU’s priorities around decarbonising H&C. Its amendments reflect new priorities in relation to the role of RECs including:

- Member States are to encourage local and regional administrative bodies to include renewable heating and cooling in their city planning infrastructure and consult network operators to reflect on the impact of demand-response and efficiency programmes (Article 24[8]).
- Renewable-based district heating and cooling networks shall be promoted by RECs through regulatory measures, financing arrangements and support (Article 22).
- There shall be the creation of a coordination framework between district heating and cooling network operators and potential sources of waste heat and cold to facilitate the use of waste heat and cold. Said coordination will ensure dialogue with energy system actors including DSOs, industrial sector enterprises, science experts, local authorities and RECs (Article 24[4]).

The current legal landscape in the EU for district heat is dominated by Article 24 [4] in RED-II which seeks to strengthen the district heat target for the EU by 2030. This obliges Member States to endeavour to increase the share of RES in their H&C sectors by granting producers of renewable heat or waste heat access to the grid (Third-Party Access (TPA)) [13]. District heating and cooling has been put at the epicentre of the amendments in the Recast for RED, has included new provisions for district heating and cooling in Article 23[4] and Article 24[1] and 24[4] and [5]. Furthermore, final consumer prices and efficient and sustainable technologies for district heat are currently regulated under Recital 18 of the amended Energy Efficiency Directive (EU) 2018/2002 (2018) [33]. However, not all Member States have complied with this.

Despite the above, it is unclear where TECs map onto the two legal categories set out by RED-II and IEMD. While the definition for RECs under RED-III clearly allows for the provision of thermal energy, the extent to which CECs can engage in similar activities under the IEMD is unclear. On the one hand, the IEMD was designed to re-organise the power grid, and as a result the activities of CECs can be read as limited to the electricity sector, thereby excluding heat. While the IEMD allows for CECs to deliver energy efficiency services, it also allows CECs to offer ‘other’ energy services [34].

Community-based district heat introduces unique challenges that differ from the nature of electricity-based energy communities. In a district heat-TEC, members must be physically connected to the same thermal pipe network – unlike electricity counterparts that can virtually share energy. Furthermore, the fuels used for district heat may further muddy the legal waters for categorising TECs – specifically where a district heat uses waste heat, which does not always qualify as ‘renewable’ (but is treated as a compliance equivalent for meeting EU targets).

While the legal definitions of the IEMD and RED II provide a framework for market participation, TECs are fundamentally distinct from their electricity-based counterparts due to their deep spatial embeddedness. Unlike electricity, which can be shared virtually across a national grid, thermal energy is a “spatial prisoner” [35], constrained by the physics of heat loss and the high capital costs of underground piping. Consequently, the viability of a TEC is not merely a matter of governance but is contingent upon linear heat density and local settlement patterns.

Literature in spatial energy planning highlights that district heat networks are subject to path dependency created by legacy infrastructure [36]. High-temperature legacy pipes often present a technical mismatch for the low-temperature renewable sources typically favoured by RECs. Furthermore, the spatial distribution of waste heat is often concentrated in industrial “point-sources,” creating a geographical radius of viability that dictates the community’s membership boundaries [36]. This physical reality necessitates a move beyond market-centric analysis toward Integrated Urban Energy Planning. In this context, the municipality acts not just as a stakeholder but as a “spatial gatekeeper,” utilising mapping and zoning competencies to de-risk investments and coordinate the built environment with community energy goals [37].

In line with the new EU priorities around heating, recent scholarship underscores that the success of TECs and community-owned heating networks depends less on technology choice than on governance, institutional frameworks, and citizen participation. [20] show how German TECs thrive when municipalities provide political support and professional expertise, highlighting that strong community engagement is indispensable given the high customer base needed for district heating to be viable. Similarly [38], demonstrate through agent-based modelling that institutional enablers, such as training community boards and subsidy allocation, outweigh purely technical considerations in shaping TEC formation, stressing the importance of balanced decision-making criteria for long-term success.

Despite the growing body of literature on electricity-based energy communities, the governance and regulatory conditions enabling the formation and operation of thermal energy communities remain substantially under-studied. While insights from electricity-focused scholarship – including work on cooperative governance, prosumerism, and regulatory enabling frameworks – offer valuable conceptual grounding, they do not translate directly to the thermal context. The spatial embeddedness of heat, the high capital requirements of underground pipe infrastructure, the distinct regulatory treatment of heating under EU law, and the natural monopoly characteristics of district heating networks together constitute a qualitatively different governance challenge that existing energy community literature has not adequately addressed. [39] identified this as an emerging research gap, and subsequent scholarship on TECs – including work on German cooperative heating models [20] and institutional enablers of TEC formation [34, 38] – has begun to address specific dimensions of this gap without producing a systematic account of the enabling regulatory and governance conditions at EU level. This paper responds to that gap by drawing on the Danish case to develop transferrable middle-range insights into the legal, financial, and institutional arrangements that enable community-based actors to participate in district heat systems – contributing to an emerging framework for the governance of thermal energy communities within liberalised European energy markets.

The above background establishes the legal and regulatory context for energy communities in EU law, the distinct governance challenges posed by thermal energy communities relative to their electricity-based counterparts, and the gap in existing literature concerning the conditions under which community-based actors can be effectively integrated into district heating systems. It is in response to this gap that the present paper examines the Danish case as a source of transferable governance lessons. The aim is not to present Denmark as a universally replicable model, but to identify the specific legal, financial, and institutional conditions that have enabled cooperative and community-based participation in district heating - and to assess which of these conditions may inform regulatory reform at national and EU level.

## Methodology

### Case study: Denmark

Denmark was chosen as the case study for several reasons. Firstly, it has been successful in decarbonising heat due to path dependencies [40]. The comprehensive infrastructure planning in Denmark has resulted in cost-effective district heat networks [41]. Secondly, Denmark ranked first in the Energy Trilemma Index (2024), indicating a balanced and robust energy system [42]. In 2022, two-thirds of Danish households received their heat from district heating. In the same year, almost 70% of district heat was produced by renewable energy - according to the annual data from the Danish Energy Agency [43] – with renewable energy including solar, geothermal, biomass (straw, wood, waste), biogas and heat pumps [44, 45]. Cooperative culture and bottom-up innovation have played a key role in the socio-technical deployment of RES deployment [19]. Further, district heat networks play a crucial role in achieving Danish net-zero goals [44, 46–49]. In 2021, 65.8% of district heat was produced using electricity [50]. The choice of case studies enables researchers to assess how differences in institutional set-up may impact the levels of policy responsiveness in the heat transition. Consistent with established case study methodology, the purpose of this analysis is not to produce statistically generalisable findings, but to develop transferable, middle-range insights into institutional and regulatory dynamics that shape decarbonisation pathways in district heating systems across different contexts [51, 52]. The study illustrates how historically embedded heating infrastructures can be progressively reconfigured through governance, ownership, and legal reforms — offering policy-relevant lessons for other EU Member States with diverse urban forms, climates, and resource endowments.

The transferability of the Danish case rests on several grounds that are worth making explicit. First, Denmark’s expansion of district heating was not primarily achieved through greenfield development but through the progressive connection of existing residential and urban stock - a context that is directly analogous to the challenge facing most European Member States, where heat transitions must be delivered within, rather than alongside, established built environments. Second, the governance and regulatory lessons drawn from Denmark are not climate dependent. They concern institutional design rather than technical performance and are therefore applicable across a range of Northern and Central European contexts with broadly comparable heating demand profiles. Member States in Southern Europe, where lower heating demand density may affect the economic viability of district heating networks, will need to assess the relevance of these lessons more carefully; the paper acknowledges this limitation and does not claim universal applicability. Third, the conditions under which Danish lessons are most likely to transfer can be identified with reasonable precision: high urban density, existing or planned district heating infrastructure, municipal governance capacity, and a legislative environment aligned with the obligations of RED III and the Energy Efficiency Directive. These shared circumstances, rather than climate or geography alone, provide the basis for the paper’s policy recommendations at EU level.

### Data collection: Rapid evidence assessment

To analyse the policy frameworks that enabled the uptake of cooperative, decarbonised district heating in Denmark, this study employs a Rapid Evidence Assessment (REA) - a structured, transparent, and replicable approach to synthesising evidence that is well suited to governance, legal, and institutional research questions [53–55]. Situated between narrative literature reviews and full systematic reviews, the REA is particularly appropriate for policy-oriented research where timely synthesis is required without sacrificing methodological rigour. The choice of this method directly reflects the study’s aim to examine how legal, regulatory, and governance frameworks enable the integration of energy communities into district heating systems, rather than to evaluate technical performance or infrastructure design. A full systematic review, while more comprehensive, was beyond the resources available within the study’s timeframe; the REA was therefore the most rigorous method proportionate to the study’s scope.

The REA aims to answer the following question: What laws and policies have been implemented in Denmark, and how have these enabled the rapid expansion of cooperative-owned district heating? The following search strings were used:

- (“district heat*” OR “heat networks”) AND (Denmark OR Europe) AND (policy OR regulation OR governance OR law OR framework).
- (“district heat*” OR “heat networks”) AND (cooperative OR “community-owned” OR “municipality-owned” OR “energy communities”) AND Denmark.
- (“community energy” OR “energy community” OR “cooperative energy”) AND (district heat* OR “heat networks”) AND Denmark.
- (“municipality” OR “local government”) AND (district heating OR “heat supply”) AND (policy OR regulation OR governance).
- (“district heat*” OR “heat networks”) AND (“European Union” OR “Europe”) AND (policy OR regulation OR governance).

Evidence was screened against the following inclusion criteria: geographic scope limited to Europe and Denmark; reference to socio-technical policies, laws, and regulations; reference to outcomes related to district heating expansion or end-user inclusion; publication between 2017 and 2025, with earlier sources included only where theoretically relevant; peer-reviewed academic literature; and grey literature produced by governmental or institutional bodies. Sources were excluded if published in a language other than English or if employing quantitative or modelling-based methodology, given the study’s focus on governance and policy mechanisms.

The screening process followed PRISMA methodology [56]. Of 2,546 titles initially screened, 351 abstracts were assessed for relevance and stored in Excel. From these, 92 documents were selected for full-text review, of which 18 were duplicates and 7 were inaccessible. A total of 71 sources were included following snowballing and citation searches. The final assessment comprised 28 peer-reviewed articles and 19 grey literature sources, stored in Zotero. Evidence was critically appraised for relevance and robustness in line with REA standards [55].

**Fig. 1:**
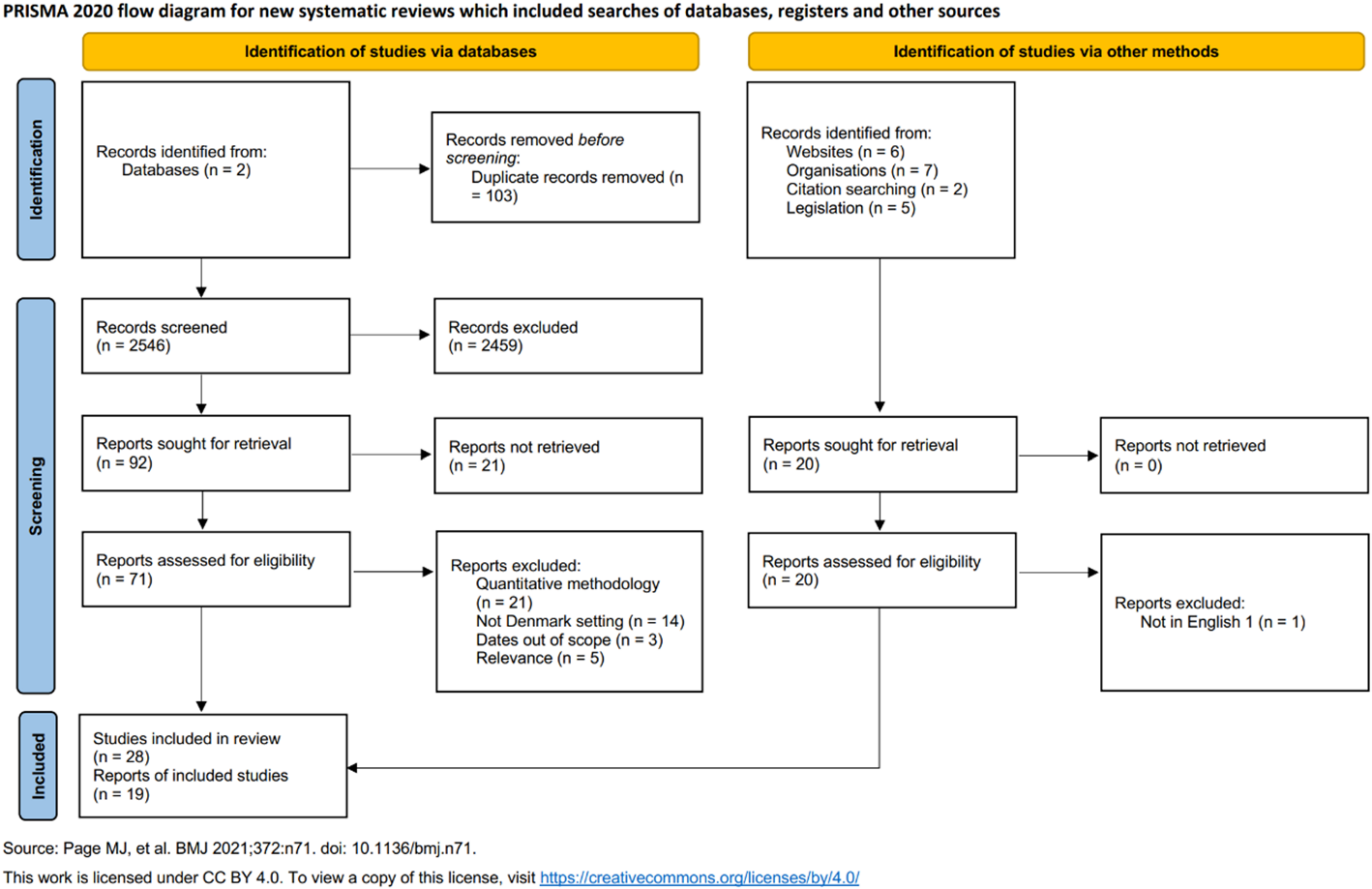
PRISMA 2020 Flow Diagram for New Systematic Reviews based on the PRISMA 2020 updated guidelines [56]

Several limitations and potential sources of bias should be acknowledged in interpreting these findings. The selection of Denmark as a single, high-performing case may favour positive lessons and under-represent the implementation challenges encountered in less mature systems. The exclusion of quantitative and modelling-based sources, while analytically coherent with the study’s governance focus, may under-represent techno-economic considerations that also shape district heating viability. Finally, the restriction to English-language material may under-represent domestic Danish practitioner knowledge. These trade-offs were accepted to maintain analytical coherence, and their implications are considered throughout the discussion of findings.

## Results and discussion

The background presented in Section 2 demonstrates the limitations of the law and the literature to electricity. To this end, the section below presents the review of the evidence collected from Denmark – as a success story in integrating cooperative and energy community ownership in the district heat sector.

The literature confirms that Denmark’s regulatory strides have resulted in the successful and large-scale uptake of district heat in the country, enabling the decarbonisation of heat. However, authors and studies attribute different weight to the factors that enabled the expansion of district heat. In this light, this section presents the results of the Rapid Evidence Assessment in an attempt to map out what regulatory and supporting policy reforms are required to integrate energy communities into the planning and operation of district heat networks in line with EU climate targets – by evaluating how this has been done in Denmark as a successful case study.

Key themes arise in the context of this Rapid Evidence Assessment. These themes and policies can be translated, broadly in principles that have underscored the policy and regulatory framework driving the expansion of district heat in Denmark. Namely, these are:

1. Rule of law via the Heat Supply Act (1979);

2. Municipal heat planning powers;

3. Non-profit rules;

4. Cooperative and municipal ownership (end-user participation).

Some insights that are not as obvious or as obviously analysed in the evidence is the role of strong institutional bodies such as the Danish Energy Agency’s role as a facilitator of the transition. However, it is clear that the heat transition in Denmark has fostered strong multi-actor governance foundations. Furthermore, while the nature of district heat networks fundamentally distinguishes them from electricity or individual home heating appliances due to their spatial embeddedness, the physical characteristics of the technology were not homogenously emphasised as a deciding factor for its expansion throughout the literature assessed in this Rapid Evidence Assessment. As most of the evidence focused on policy and regulatory mechanisms for expanding district heat, the studies seemed blind to the infrastructural challenges associated with the implementation and expansion of district heat as a mainstream source of urban heat – which can be identified as a limitation of this study.

Nonetheless, Denmark’s rapid expansion of district heat and heat decarbonisation offers key lessons for other nations as a result of the policies and regulations that have resulted in the decentralisation of heat governance. Below is a synthesis of policies, their impacts, and transferable insights, structured by policy type and enabling factors, as identified by the academic and grey literature with the aim of supporting the integration of energy communities in urban district heat networks in the EU.

### Rule of law

A key foundation to energy policy is the rule of law. Denmark’s heat transition and the district heat sector are regulated by the Heat Supply Act (1979 - revised in 1990, 2000 and 2005 (HSA)). The HSA has been recognised by almost all the evidence assessed in this Rapid Evidence Assessment as a cornerstone of the expansion of district heat in the country. However, different authors have attributed various importance to the role of the HSA – from introducing district heat to scaling it once successfully implemented. The HSA’s objective is “*to promote the most socio-economic (including environmentally friendly) use of energy to heat buildings and provide hot water*,* and within this framework to minimise the energy supply dependency on fossil fuels” (§ 1)* [57, 58]. The ownership and price regulation models under the Danish Act have prioritised consumer welfare through several initiatives. In relation to national law and domestic conditions, the Danish model for energy communities in district heat has allowed small-scale deployment of district heat in rural areas, and the municipal ownership of district heat networks in urban areas. As a result, district heat coverage grew from 30% to 65% of households by 2020 [22].

The Act provided the legal foundation for mandatory connection policies until 2019. On the one hand, at the face of it this may seem unreasonably authoritarian. Indeed, removing consumer choice and autonomy has been widely analysed in the literature, particularly in the context of mandating connection for what is a natural monopoly. However, mandatory connection policies have been based on *socio-economic impact –* a key principle that underscored the Danish success - ensuring a stable customer base, reducing investment risks [22, 59]. This was highlighted in [60]’s work, which showed that Danish households had the most positive perception of district heat, despite having some of the most stringent regulatory regimes. While households in countries without liberalised price regulations did not show a positive perception of district heat, Denmark was the exception – owing largely due to the reliability of heat provision and the public ownership structures that enable these. Adding to this, other authors have attempted to link the successful example of the Rule of Law in Denmark to the technical characteristics of district heat. [61] specifically argued that in contrast to RES, the technical characteristics of district heat – such as its lifetime and the linear relationship between size and consumers – influences its resilient^1^ and consistent presence in Denmark. However, akin to other works, the authors conclude that the technical characteristics of district heat are in fact complementary to the strong governance mechanisms in place that expanded district heat in the country.

The consistency of the Heat Supply Act as a predictable legislative framework for district heat has been widely recognised as one of the key success factors for the expansion of district heating in the country. Some authors have described this as a “long, stable, and robust regulation” [57] which fostered a favourable environment for the development of cooperative-owned district heat, protecting consumers and making district heat competitive. [62]’s research comparing historical heat transitions in Denmark, the UK and the Netherlands, showed that government intervention in public ownership of distribution infrastructure played a key role in expanding new heat systems. Importantly, it is highlighted that the development of new actor roles and regulations were imperative in pushing the respective heat transitions in all countries. The authors explain that competition, liberalization and market forces were not sufficient for the expansion of large-scale infrastructure. Instead, these were built through collective efforts and long-term state planning.

As the 1973 oil crisis propelled the need for concentrated action in Denmark, the HSA has prioritised establishing energy security in the country. Whilst it has been identified that motivations between district heat expansion vary and may today be defined (in part) by environmental concerns, the HSA’s original objectives included the integration of natural gas from the North Sea in the Danish district heat mix. Unlike other authors who do not discuss the role of natural gas from the North Sea [19], ’s historical account highlighted this, and how tax revenues from natural gas projects were used to support the Danish welfare state. These priorities were later changed, with the 2000 Climate Policy, which sought to increase the share of RES in district heat in the country [63]. The above shows that the HSA's enduring relevance lies in its specificity for its original objectives as well as its function as a stable and adaptive legal architecture capable of accommodating shifting energy priorities without undermining the governance foundations it established. 

### The role of municipalities

Furthermore, the HSA delegated strong powers to municipalities in the country, which have been identified as a key policy for integrating local initiatives into broader infrastructure strategies [64, 65]. The amendments to the Act in 1990 decentralised heat planning – delegating powers to municipalities for infrastructure planning. The aim was to increase municipal ownership and to facilitate the integration of local initiatives in municipal heat planning practices [66]. The ‘tools’ in the HSA toolbox included: law on heat supply, planning law, and environmental and building laws [67]. Municipalities were responsible for the implementation of mandatory connections in the entire locality or selected parts of it. A key governance pillar for regulating district heat in Denmark is the role of municipalities in heat planning and zoning. This mandatory requirement of local authorities has been identified as a key driver for Danish success not only in the academic literature, but also in other sources reviewed [15]. More recently, the Danish Heat Law (2024) set out the roles and responsibilities for local authorities with regards to planning, zoning, and decision-making powers, including limits on approving new fossil gas and biomass heating. In support of this, the Danish Energy Agency provides methodologies and socio-economic input data for municipal heat planning processes through technology catalogues and offers support. Further, planning processes are financed using municipal budgets, while the Danish Energy Agency also provides dedicated financial support programmes for consultancy services and project implementation [68].

Under Danish legislation, the municipal council (elected politicians) must cooperate with utilities to plan the heat supply of the municipality [67] – ultimately resulting in multi-actor governance structures [57]. As a result, there are 400 district heat companies in Denmark, with 50 of them owned by municipalities, supplying approximately 50% of all district heat. 250 cooperatives approximately 50% of district heat, and 10 private companies provide less than 1% of heating [67]. In comparative studies, municipalities have been critical for the success of heat planning and implementation – using a plethora of similar, and different, strategies to expand district heat. Some posit that the paradigm shift has downplayed the role of governments as drivers for the energy transition [69], enabling long-lasting strategic energy planning [19]. [63] ’s assessment of short-term heat transitions argues that the increased focus on energy security as a result of the 1973 – and the origins of the HSA – resulted in radical restructuring, with the decentralisation of planning powers achieving these energy security objectives.

Importantly, it is identified that municipalities were not alone in this process – making the implementation more transparent and less arbitrary. [65] have argued that the centralised existence of market standards and guidelines for district heat development played a critical role in improving the compatibility of district heat system components, ultimately resulting in price efficiency. This not only resulted in effective local planning, but also the export of district heat technologies internationally – with companies such as Danfoss and Logstor – registering a shift in R&D (Ibid).

Other accounts have been more hesitant to praise the decentralisation of heat planning for various reasons. Earlier work on municipal planning has shown that the decentralised nature of the Danish model introduces a potential scale mismatch between local administrative boundaries and regional system efficiency. Within the framework of Smart Energy Systems, district heating is not merely a local utility but a provider of flexibility for the national power grid through sector coupling [70, 71]. If planning is optimised solely at the municipal level, there is a risk of sub-optimisation where local interests - such as prioritising a specific local biomass source - may conflict with broader regional goals like the integration of large-scale industrial waste heat or national electricity balancing [72].

This tension highlights a significant barrier to transferability: while Denmark benefits from high 'institutional thickness' and technical expertise within its municipalities, many other European contexts exhibit a 'capacity gap' [73]. In regions with weaker municipal competencies, a purely bottom-up approach to heat planning can result in fragmented, inefficient networks that lack the scale to interact effectively with the national energy landscape [74]. Thus, while Denmark demonstrates the power of local engagement, the literature suggests that its success is contingent upon a delicate balance between local autonomy and a strong national framework that ensures regional technical coherence.

Furthermore, more recently, it has been argued that infrastructure planning priorities and decisions have been “far from optimal”[66]. Johansen and Upham have argued that this has led to *de facto* competition between municipalities, resulting in sub-optimal regional infrastructure planning [66]. Ultimately, the authors argue that the approaches to heat supply are ideological and political, emphasising the lack of bottom-up local knowledge and participation in Danish energy planning. More specifically, in contrast to this criticism, comparing strategic energy plans from 98 Danish municipalities [64], argued that spatial planning is distinguished by strong vertical coherence and aligns urban development between national, municipal and community levels. However, closer to the tune of [66]’s criticisms, the author found that there is a lack of strategic and long-term orientation of municipal heat planning, which when looked across multiple municipalities, results in incoherent strategies.

This comes in sharp contrast with other authors’ findings which suggest that the large uptake of TECs, and the delegation of municipal powers, paired with socio-economically and environmentally informed planning has resulted in a successful localization of heating powers [60, 62, 65, 75, 76]. A few points of criticism can be raised against [66]’s propositions. First, one would argue that an optimum can only be achieved via machine-based decision-making. Any decision that has included a human factor, may not be ‘optimal’. Furthermore, their account criticises the politicization of municipal ownership and planning decisions. However, a counterargument can be made that the inclusion of municipalities in district heat ownership and planning can be illustrative of ‘energy democracy’ – a term with contentious definitions in the literature. Perhaps, an example closer to Denmark is that of “Innovative Democracy” – a term coined to describe the political process related to the energy transition, occurring when the political process related to the energy transition is influenced by market-dependent actors (such as consumers, municipal cooperatives etc.), and particularly, by market independent actors such as NGOs and the public debate. These, together with associations, advocate for their private interests in the energy transition [61, 77–79].

The multi-actor governance, enabled by the HSA and built on by municipalities in Denmark, has been identified as a key component to the expansion of district heating the country. Salite and colleagues, highlight that the regulatory environment and wide dissemination of information resulted in cooperation between multiple actors in district heat systems, ultimately derisking investment in district heat and driving down prices [65]. This has also been reflected in Herreras Martínez and colleagues's findings – where participants emphasised that the competition is not amongst cooperatives or municipalities, but rather between technologies, due to transparent information sharing, which has led to fertile grounds for the development of district heat [57]. This was further confirmed in earlier study by [62], who showed that the inclusion of public and private actors probed important in moving infrastructural developments forward. Taking a governance perspective [63], ‘s comparative analysis of global heat transitions put the emphasis on polycentricism. They argue that harnessing the power of diverse perspectives, polycentricism results in cooperation rather than competition. They describe the value of this from a problem-solving lens - that multiple actors review the same problem, producing a variety of potential solutions and better experimentation.

The above demonstrate of the importance of strong inter-institutional collaboration on the success of heat planning particularly in the attempts to decentralize and decarbonize heat networks. By fostering localised, cooperative heat systems within cities advance decarbonisation and equity goals while enhancing urban resilience - insulating communities from geopolitical energy shocks and fuel price volatility.

### Financial incentives

Adding to the impacts of the rule of law on the development of district heat in Denmark, authors in the literature have also stressed the importance of financial incentives for the rapid expansion of district heat in the country – particularly in the technology’s marketability and expansion. Some identified long-term, low-interest loans, such as via the Municipal Credit Bank, and subsidies for district heat infrastructure as key instruments in this regard [57, 63]. Furthermore, tax policies had district heat more competitive in contrast to its other counterparts [63, 80, 81]. Incentives such as the reduced electricity tax and a 30% investment subsidy for heat pumps in district heat, along regulatory constraints for fossil fuels have resulted in the shift decarbonisation of district heat [82]. This has resulted in a doubling of power to heat electric capacities from 232 MW in 2014 to 542 MW in 2021 – resulting in large-scale sector coupling. Furthermore, the stable policy environment for district heat in the country resulted in standardised financing procedures, derisking investment in district heat. In part due to the mandatory connection rule, and in part due to other financial and fiscal measures [57].

When assessed in tandem with the cooperative and municipal ownership of district heat, this becomes an important characteristic to the growth of a market that is not allowed to make a profit. Long-term concessional bank loans secured by municipal guarantees, equity capital and subsidies played an important role in developing district heat in the country – a finding validated in the literature such as in [65], but also via stakeholder interviews, such as in [57]. In the latter, interviewees outlined the importance of the socio-economic methodology in the effectiveness of this, and that municipal guarantees signalled to the market confidence in the technology and the projects, resulting in no cooperative going bankrupt - and, therefore, the guarantees never being used in practice [57]. 

Nonetheless, some have downplayed the role of financial incentives in expanding district heat – attributing greater importance to governance structures. [80] conclude that different triggers can result in different transition patterns – for instance, moving away from an ‘opportunity’ narrative to a ‘problem’ narrative (as in the Danish case) led to more expansive alternative pathways. The authors suggest that traditional policy tools such as public investment, subsidies, tax-based incentives, regulation and planning, are important in later transition phases rather than for the establishment of district heat. Instead, they argue, the Danes first set out the ‘preconditions’ for an expanded district heat network, which was then consolidated via more “coercive” policies (such as mandatory connections) to accelerate the transition in different local contexts. Taken together, the evidence suggests that financial incentives functioned most effectively as consolidating instruments that reinforced governance and ownership structures that had already established the institutional conditions for district heat expansion.

### Non-profit principle

The third characteristic of success, mandated by the HSA, has been the non-profit principle, which, paired with the cooperative and municipal ownership, has ensured affordability and reinvestment in infrastructure [57, 59, 60, 65, 67, 67, 83].

Danish price regulation stipulates that district heat is operated under the true-cost principle. Production and network companies are, therefore, regulated under the non-profit principle. The non-profit principle came to complement the mandatory connections in the country, leading to strong consumer protection, as well as the expansion of cooperative ownership therein [84]. It is argued that paired with mandatory connections, the non-profit principle mitigated the risks associated with an insufficient customer base, and increased investment in infrastructure [85]. However, with the mandatory connections discontinued in 2019, the efficacy of the principle, will need to be re-assessed to validate this finding in a changing context. Despite this, given the widespread implementation of district heat, it may be the case that mandatory connections are simply no longer necessary, ultimately leading to the need for non-profit principle standing as the key consumer protection mechanism for locked-in consumers. However, the principle does not guarantee low prices for consumers and efficient solutions for all district heat networks as it is vulnerable to local mismanagement [86]. This highlights the need for regulatory standards with clear consequences for noncompliance. In Denmark, the true-cost principle has worked well in part because it has been combined with widespread cooperative ownership - see below.

As district heat networks remain natural monopolies, price regulation must account for local availability. [86]'s review of regulatory regimes for district heat networks proposes that a mix of price-setting regimes and ownership models is appropriate. Another example proposed is joint finance – for instance, where local government contributes with equity in the form of land and assets. However, it has been identified that private enterprises take fewer risks, resulting in smaller rates of return and the reduced incentive for long-term efficiencies under a joint finance regime. In Denmark, commercial investors require 8% or more as an internal rate of return, while consumer-owned or municipally owned district heat companies require lower returns to make ends meet as their purpose is to provide affordable and locally controlled heat solutions (p.134). 

By contrast, [87]’s comparative study of district heat networks contends that the introduction of competition on the supply side is more appropriate. Indeed, the issue of competition prevails in the literature [65, 83]. As district heat is regulated by the non-profit principle, the heavy subsidisation of heat pumps incentivises alternatives to district heat for locals when they transition from oil and gas boilers [83] – leaving the sector vulnerable to transitions to individual heat systems. This is further confirmed by [65], whose research shows that the non-profit principle does not incentivise district heat companies to be economically efficient, holding back new district heat companies or new owners in existing companies. This was reiterated in [85]’s work, who showed that the limited pressure to improve productive efficiency due to the non-profit principle implicates the role of price regulation on heat pricing. One way that the Danish Utility Regulator has attempted to mitigate this is via voluntary benchmarking and transparent complaint processes, but this has not been effective. Indeed, the application of the non-profit principle in Denmark, has been identified by one interviewee as a progressive way of seeing district heat as an asset akin to “freshwater” [83]. In light of this [85], suggest that the ownership of district heat companies plays a greater role in influencing pricing and transparency in contrast to price regulation. The non-profit principle represents more than a regulatory mechanism for cost control; it embodies a governance approach that redefines heat as an urban public good. By embedding affordability, transparency, and community reinvestment into the operation of district heat networks, the Danish model demonstrates how local, cooperative, and municipal ownership can anchor social equity within the decarbonisation of urban infrastructures. As cities across Europe confront the intertwined challenges of energy poverty, climate risk, and resource scarcity, such frameworks offer a pathway toward resilient, citizen-centred, and circular urban energy systems - core ambitions for the urban futures of a just and climate-compatible Europe.

### Consumer ownership

Going hand-in-hand with municipal ownership and the non-profit principle, cooperative ownership structures ensure end-user participation in energy decision-making. Adding strength to the consumer protections discussed above, consumer cooperatives and municipal ownership have been identified as another core factor for the success of district heat expansion in Denmark as these ownership structures have fostered trust and local engagement in the heat transition in the country [60, 61].

The Danish cooperative ownership archetype has remained stable through different crises. This has been largely the case due to financial incentives, and the role of the non-profit principle, which deterred private firms from investing in district heat in the country [59]. Whilst it may be argued that cooperative structures are what necessitated the development of the non-profit principle, others argue that the uptake of cooperative ownership is the direct result of the non-profit principle [61]. Research in consumer perception of district heat systems has shown that countries with public ownership of district heat have more positive perceptions, as consumers also perceive their district heat prices to be lower compared to other groups [60]. In the literature, experts interviewed pointed out that a consumer-owned district heat business model is one of the main reasons for the Danish district heat success, because consumers are proud of their company, being able to run it and to influence its decisions [65].

When contrasted to other countries with large numbers of historical TECs (Germany and the Netherlands), [85]’s work showed that Denmark’s HSA played a key role in fostering the expansion of district heat by mitigating evolving policy landscapes, limited public familiarity and financial constraints. The HSA embeds participatory governance directly into Denmark’s heat sector by requiring that most district heat company boards be elected by consumers or municipal representatives [57]. This structure grants citizens both democratic oversight and first-purchase rights in the event of company sales, ensuring that ownership remains rooted in local control [61]. Empirical studies highlight that consumer representation on utility boards has been instrumental in sustaining public trust and household support during the heat transition. Furthermore, mechanisms of accountability, such as the right of consumers to dismiss cooperative boards, have reinforced transparency and responsiveness within the system [85]. A notable example is the 2008 case in which residents and the municipality repurchased district heat assets from E.ON after concerns about opaque pricing - illustrating how local agency can reclaim essential infrastructure in the public interest [63, 85]. Like [63, 80] argue that the transition benefited from coordination between the government and other organizational entities – whereby cooperatives managing district heat were sometimes being directly run by local governments, which was key for reducing the cost of district heat for consumers. In this regard, the authors conclude that the HSA enabled the expansion of district heat via decisive and consistent acceleration since 1979. This, paired with different governance styles and differing roles for users, also showed that there are multiple options for social buy-in to a new system.

When compared to other countries with large district heat markets - such as Sweden - Denmark’s governance model grants greater consumer influence through statutory requirements for local board representation and decision-making [88]. This participatory foundation has contributed to the expansion of integrated heat networks across multiple municipalities, reflecting how democratic control can underpin coordinated infrastructural development. However, recent research reveals a decline in civic engagement with utilities: professionals report limited public interest in serving on boards, while many perceive residents merely as passive consumers rather than as partners in co-producing flexible, future-ready heat systems. This disconnection between technical professionals [66, 76] and citizens risks constraining the adaptive potential of district heat networks - particularly as digitalisation and automation redefine the role of users within increasingly intelligent energy systems [88]. For instance, work by [76] showed an innate assumption amongst district heat professionals that residents are perceived as passive recipients of information and technology services – rather than active market actors. This finding hints at consumers’ changing agency. To this end, Andersen et al. [77], suggest that designing diverse resident engagement formats via co-creation may solicit engagement rather than acceptance – moving closer to the early late 1990 s Danish archetype of cooperative ownership.

Municipalities and cooperatives continue to play a vital role in bridging this divide by facilitating information-sharing, demonstration projects, and participatory planning. Still, the relationship between providers and consumers must evolve from a transactional one to a collaborative governance model that recognizes end-users as both *market participants* and *political constituents*. As several scholars note, Danish policy design has historically relied on public legitimacy - earned through affordability, reliability, and strong consumer protection - to justify mandatory connection schemes. In this way, users’ political acceptance of collective heat systems was anchored in socio-economic fairness and environmental rationale, generating long-term confidence and social license for district heat expansion [57, 60, 63, 80]. Seen through the lens of Urban Futures, consumer ownership and participation in district heating exemplify a broader shift toward urban energy citizenship. Such participatory models redefine cities as spaces of democratic experimentation, where social equity, climate resilience, and system innovation converge. Strengthening these urban energy commons can therefore be understood as a critical pathway to decarbonized, inclusive, and resilient cities of the future.

### A generalisable benchmark or a structural outlier?

A few arguments may be made to place Denmark not simply as a best practice, but as an outlier with structural preconditions that may limit the case study’s replicability to other Member States.

First, it has been argued in the sociotechnical transitions literature that the transition to district heat was a crisis-driven one due to the 1973 oil crisis. While this did not automatically result in the choice of district heat, [80] have argued that the weakness of incumbent regimes (such as oil-based electricity), and weakness of alternatives (nuclear) due to public residence ultimately led to Denmark favouring district heat. This was reiterated by [19] who argued that the public rejections of nuclear energy played a role in the uptake of district heat, and that this transition did not happen in a vacuum.

Furthermore, authors point out that the Danish socio-technical development has been defined by a collective culture of cooperation, informed by “collective societal memories of energy resource scarcity” which have further been enabled by public government trust and support – a cultural model that may not be easily replicable in other jurisdictions [19]. The argument has been made that the Danish heat supply sector is informed by underpinnings of cooperative culture and welfare state values, which guided the sector and mirrored societal foci and challenges [66]. Indeed, a comparative study between Denmark and Sweden showed that while both countries introduced policies promoting district heat and security of supply, Danish heating was dominated by cooperative and municipal ownership (to date), whereas Swedish heat was owned by municipalities until 1995, which was been attributed research to cultural dissimilarities between the two countries [85]. Specifically, regarding cooperative ownership for district heat, interviewees in [57] have argued that the tradition of cooperative ownership in the agricultural and electricity sectors translated to the district heat sector, resulting in the expansion of local heat supply and governance after the oil crisis [57]. [79] has also argued that sociocultural characteristics have played an important role in political processes towards radically new technologies and the proximity between “the legislators and the legislated”, which the authors attribute to the trend of Innovative Democracy in the country.

However, this has been a culture developed over a period of 30 to 40 years – a period that other European Member States also must move beyond a traditionally opaque and oligopolistic market, to one that engages end-users as agents of the transition. The decarbonisation of the European Union’s heating sector remains a critical yet underdeveloped dimension of its broader climate governance framework. Despite the clear legal and normative commitments under the European Green Deal and the ‘Fit for 55’ package, current legislative instruments insufficiently integrate energy communities into the governance of district heat networks. This lacuna reflects a broader oversight in EU climate and energy law: the underutilisation of decentralised, participatory legal forms that can advance the principles of subsidiarity, energy justice, and democratic legitimacy.

Recognising the cultural and historical specificities of the Danish transition does not imply that its outcomes are culturally predetermined or non-transferable. Rather, Denmark’s experience illustrates how culturally contingent factors - such as high levels of social trust, cooperative traditions, and proximity between citizens and public authorities - interacted with, but did not substitute for, a set of institutionally transferable mechanisms. These include long-term legal and regulatory stability, the non-profit orientation of district heat operators, strong municipal planning mandates, access to municipal guarantees, and clear public-interest obligations embedded in heat supply law. While cooperative ownership may have been facilitated by Denmark’s socio-cultural context, the governance architecture that enabled its durability and scalability is not inherently culture bound. Similar institutional arrangements - particularly those relating to legal certainty, local authority empowerment, and the insulation of heat networks from short-term profit maximisation - are, in principle, replicable across Member States with differing cultural traditions. From this perspective, Denmark may be understood as a best practice, demonstrating how decentralised, participatory governance models can be operationalised through law and public policy. This distinction is critical for EU-wide policy design, as it shifts the analytical focus from cultural exceptionalism to the design of institutional conditions capable of fostering energy community participation in district heat networks across diverse national contexts.

## Conclusions and lessons learnt

This paper set out to address the following research question: what regulatory and supporting policy reforms are required to integrate energy communities into the planning and operation of district heating networks in line with EU climate targets? Drawing on a Rapid Evidence Assessment of the Danish case, the findings demonstrate that effective integration depends not on technical innovation alone, but on the alignment of legal mandates, governance structures, and financial arrangements that position energy communities as legitimate actors within district heating systems. The Danish case offers a compelling model of how coherent, long-term regulatory planning - centred on public and cooperative ownership, transparent pricing, and meaningful end-user participation - can decarbonise heating infrastructure while embedding democratic governance at the local level. Denmark’s experience shows that urban heat transitions succeed when legal, financial, and governance frameworks align to empower municipalities as active agents of change rather than passive recipients of national policy. Stable legislation, transparent regulation, and participatory ownership can, in the right conditions, transform a technical utility into a social infrastructure that sustains community trust and system resilience. As European cities diversify, however, policies must evolve to engage harder-to-reach populations - including renters and low-income households - and to avoid reproducing new forms of energy dependency [63].

### Study limitations and further research

This scope of the study has been limited to households and does not include businesses (including small and medium enterprises). Nonetheless, SMEs can participate in energy communities, and as waste heat gets incorporate in district heat, it would be valuable to further assess the business models that can arise for TECs in this context. As this paper presents a Rapid Evidence Assessment and not a full systematic review, the findings are limited to a smaller number of evidence, and stricter exclusion criteria – such as the exclusion of quantitative research papers from the scope of the study. The results in this work raise implications for further research avenues. Starting from Denmark, further empirical research can be done to assess the correlation between the above-mentioned factors and the expansion of district heat in the country. Furthermore, work on the economies of scale for district heat in urban settings would add further value to build on proposing successful regulatory and planning policies in the EU. Beyond Denmark, as global temperatures rise and EU Member States face higher cooling demands, further expanding on the summer poverty research portfolio to assess the expansion of district cooling and the regulatory mechanisms to do so would add value to potentially integrating lessons learnt for district heating.

### Study implications and recommendations

Decarbonising urban heat systems lies at the heart of creating resilient, equitable, and climate-neutral cities across Europe. The evidence assessment of the Danish regulatory and legal framework reveals that the foundation of a successful district heating transition rests on three interconnected pillars: the rule of law, as underpinned by the Danish Heat Supply Act and a clear delegation of municipal heat planning powers; non-profit regulation and transparent pricing; and robust end-user participation. Together, these features enabled the Danish market to evolve beyond a conventional heat provision model into one that is democratically governed and community anchored.

The transferability of these institutional lessons is necessarily conditioned by structural factors that vary across Member States - notably local heat density, municipal governance capacity, and the pre-existing structure of national district heating markets. In jurisdictions characterised by low urban density, weak local government capacity, or highly liberalised heat sectors, direct institutional replication will be more limited. The core lesson is therefore not the wholesale adoption of Denmark’s ownership model, but the importance of aligning legal mandates, planning instruments, and regulatory incentives with local system conditions. This differentiated approach supports a realistic EU-wide policy framework that accommodates Member State heterogeneity while advancing common decarbonisation objectives. The findings also demonstrate that the risks associated with natural monopoly structures can be effectively mediated through regulatory obligations and governance mechanisms that align network operation with public-interest objectives. The policy question is therefore not whether district heating constitutes a monopoly, but how monopoly infrastructures are governed, regulated, and democratically anchored - a reframing with significant implications for EU regulatory design.

Building on these insights, the following policy levers can be drawn from the Danish experience for the broader European context.

1. Integrate energy communities into urban governance: recognise energy communities as legitimate actors within city-level energy and climate frameworks, extending EU legislation to encompass cooperative and municipal heating initiatives as part of integrated urban energy systems.

2. Mandate urban heat transition planning: require municipalities to develop legally binding, socio-economically justified heat transition plans aligned with national and EU decarbonisation targets, ensuring that heat infrastructure planning is embedded within wider urban development strategies.

3. Foster multi-level and cross-sectoral coordination: strengthen coordination between national, regional, and city-level bodies - modelled on institutions such as the Danish Energy Agency - to link local innovation with national regulatory stability and EU-wide policy coherence.

4. Empower public and cooperative ownership: support local governments through legal reform and financial incentives to establish or expand public and cooperative ownership models for district heat networks, ensuring affordability and citizen participation in urban infrastructure decisions.

5. Provide targeted financing mechanisms: direct EU and national financing - via the European Investment Bank and comparable institutions - toward city-led and cooperative energy projects, ensuring access to long-term, low-cost capital for socially inclusive and climate-aligned heat investments.

Together, these recommendations shift the paradigm of heat governance from a centralised, market-driven model toward one that positions cities as hubs of systemic innovation and social equity. Embedding energy communities and cooperative governance within the urban policy landscape does more than advance decarbonisation - it strengthens the democratic and resilience capacities of cities themselves. Denmark’s lessons offer not only a reference point for energy reform, but a pathway toward just, climate-compatible, and citizen-centred urban futures across Europe.

## Data Availability

No datasets were generated or analysed during the current study.

## Footnotes

1 Drawing on Sharifi and Yamagata’s framework [62], resilience in this context refers to the capacity of an urban energy system to withstand, adapt to, and recover from disruptions – whether technical, economic or climatic – while maintaining continuity of service and progressing to long-term decarbonisation objectives.

## Declarations

### Dual publication

No, the results/data/figures in this manuscript have not been published elsewhere, nor are they under consideration by another publisher.

### Third-party material

No, all of the material is owned by the authors and/or no permissions are required.
